# Supplementary material for: Robust Saliva-Based RNA Extraction-Free One-Step Nucleic Acid Amplification Test for Mass SARS-CoV-2 Monitoring
Source: Molecules. 2021 Oct 31;26(21):6617. doi: 10.3390/molecules26216617 (PMC8588466; doi:10.3390/molecules26216617)
Supplement: Supplementary file 1 [file molecules-26-06617-s001.zip › Rajh 2021 Saliva - v5-supplement.pdf]

Robust saliva-based RNA extraction-free one-step nucleic acid amplification test for mass SARS-CoV-2 monitoring

Extraction-free RT-qPCR *vs*. RT-LAMP for saliva

Eva Rajh [1*], Tina Šket [1*], Arne Praznik [1], Petra Sušjan [1], Alenka Šmid [4], Dunja Urbančič [4], Irena Mlinarič-Raščan [4], Polona Kogovšek [5], Tina Demšar [5], Mojca Milavec [5], Katarina Prosenc Trilar [6], Žiga Jensterle [7], Mihaela Zidarn [8], Viktorija Tomič [8], Gabriele Turel [9], Tatjana Lejko Zupanc [9], Roman Jerala [1,2], Mojca Benčina [1,2,3,#]

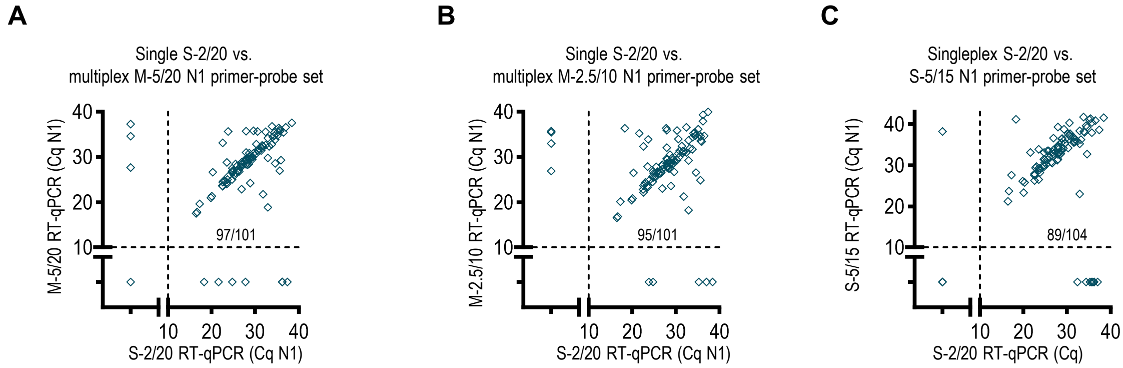

Figure S1. (**A-C**) Comparison of RT-qPCR Cq values between Cq of singleplex (S-2/20) and Cq for N1 primer-probe set of multiplex (M-5/20) (**A**), multiplex (M-2.5/10) (**B**) or singleplex (S-5/15) (**C**).

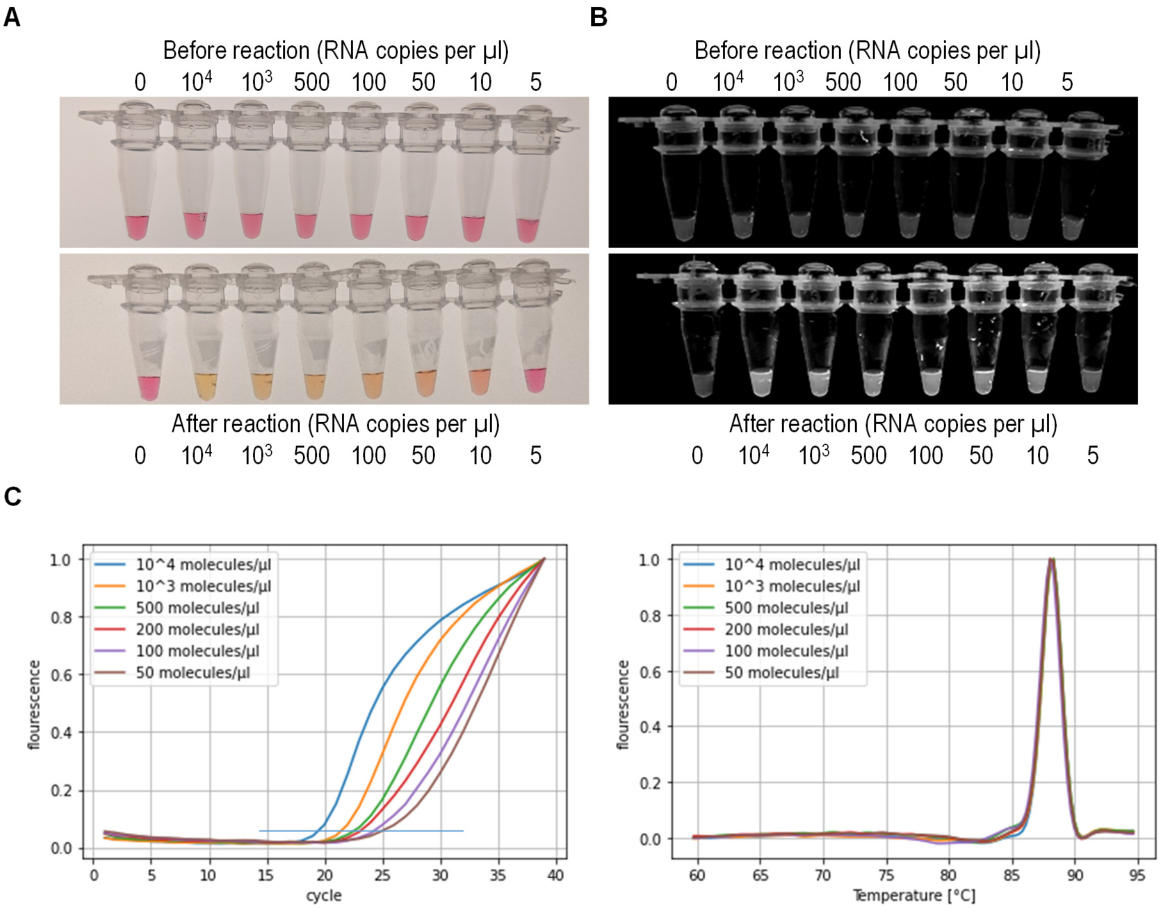

Moleculs/µl	Cq	Tp (min)	Tm (°C)
10^4	18.6	9.8	88.19
10^3	20.6	10.3	88.19
500	21.8	10.9	88.17
200	22.9	11.5	88.32
100	24.0	12.0	88.21
50	24.5	12.3	88.09

Figure S2. (**A**) Colorimetric and (**B**) fluorescence end-point detection of RT-LAMP amplicons. (**C**) Real-time fluorescence detection of RT-LAMP amplicon and melting curve. Protocol LAMP-2/20 with N2 primers set was used to amplify SARS-CoV-2 mRNA.

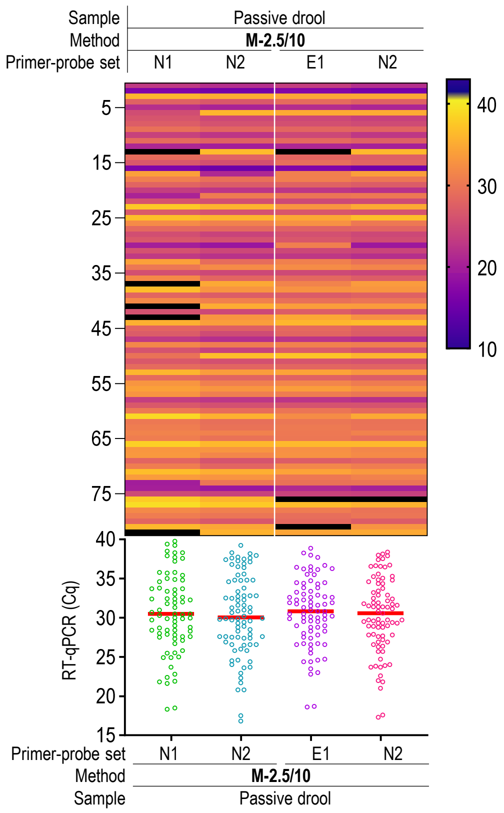

Figure S3. The heat map of matched Cq values. Pooled Cq values with depicted average value as a red line (below).

Supplementary tables

Table S1. RT-qPCR protocols

Protocol name:	Reaction volume (µl)	Sample volume (µl)	Targets	Internal control	Master mix	Equipment	Cycling conditions
**NPS**	20	5	Singleplex: E1, RdRP	human RNaP	Roche LightCycler Multiplex RNA Virus Master	RGQ PCR cycler	5 min at 55 °C<br>5 min at 95 °C<br>45 cycles: 5 s at 95 °C; 15 s at 60 °C; 15 s 75 °C
**S-2/20**	20	2	Singleplex N1	-"-	Ultraplex 1-Step 4×ToughMix	LC480	10 min at 50 °C<br>3 min at 95 °C<br>45 cycles: 3 s at 95 °C; 30 s at 55 °C
**M-5/20**	20	5	Multiplex: N1, N2	-"-	-"-	-"-	-"-
**M-2.5/10**	10	2.5	-"-	-"-	-"-	-"-	-"-
**S-5/15**	15	5 (5x diluted)	Singleplex: N1, N2	18SrRNA	AgPath-ID One-Step RT-qPCR mix	ABI7900 HT Fast	5 min at 50 °C<br>20 s at 95 °C<br>50 cycles: 15 s at 95 °C; 1 min at 60 °C

Table S2. RT-qPCR primers and probes.

Name	Sequence	Final concentration (nM)		
		RT-qPCR protocols		
		2/20; 5/20; 2.5/20	5/15	NPS
nSCoV_N1_F	GACCCCAAAATCAGCGAAAT	400	500	
nSCoV_N1_R	TCTGGTTACTGCCAGTTGAATCTG	400	500	
nSCoV_N1_P1	(HEX)ACCCCGCATTACGTTTGGTGGACC(BHQ1)	200	125	
nSCoV_N1_P2	(FAM) ACCCCGCATTACGTTTGGTGGACC(BHQ1)	200	125	
nSCoV_N2_F	TTACAAACATTGGCCGCAAA	400	500	
nSCoV_N2_R	GCGCGACATTCCGAAGAA	400	500	
nSCoV_N2_P1	(FAM)ACAATTTGCCCCCAGCGCTTCAG(BHQ1)	200	125	
nSCoV_N2_P2	(HEX)ACAATTTGCCCCCAGCGCTTCAG(BHQ1)	200	125	
h_RNaP_F	AGATTTGGACCTGCGAGCG	400	500	
h_RNaP_R	CAACTGAATAGCCAAGGTGAGC	400	500	
h_RNaP_P1	(Cy5)TTCTGACCTGAAGGCTCTGCGCG(BHQ3)	200	125	
h_RNaP_P2	(Cy5)TTCTGACCTGAAGGCTCTGCGCG(BHQ2)	200	125	
RdRP_SARSr-F	GTGARATGGTCATGTGTGGCGG			600
RdRP_SARSr-R	CARATGTTAAASACACTATTAGCATA			100
RdRP_SARSr-P2	(FAM)CAGGTGGAACCTCATCAGGAGATGC(BBQ)			100
RdRP_SARSr-P1	(FAM)CCAGGTGGWACRTCATCMGGTGATGC(BBQ)			800
E_Sarbeco_F	ACAGGTACGTTAATAGTTAATAGCGT			400
E_Sarbeco_R	ATATTGCAGCAGTACGCACACA			200
E_Sarbeco_P1	(FAM)ACACTAGCCATCCTTACTGCGCTTCG(BBQ)			400
18S rDNA_F<br>18S rDNA_R<br>18S rDNA_P	Eukaryotic 18S rRNA Endogenous Control (Applied Biosystems), sequence not disclosed			

W, A/T; R, G/A; M, A/C; S, G/C; BBQ, blackberry quencher; BHQ, black hole quencher; FAM, 6-carboxyfluorescein; HEX, hexachloro-fluorescein; Cy5, cyanine 5.

Table S3. RT-LAMP primers.

Name	Sequence	Final concentration (µM)
RNaP_B3	CTTTCCCTCATCCTTCTC	0.2
RNaP_F3	GGAGAGTGAGTTGATCAG	0.2
RNaP_LB	CAGAGGCACCTAGGATTGG	0.4
RNaP_LF	AGGCTTGCTTACCTCCAG	0.4
RNaP_BIP	TGGTGACCTGAACTAGGGTTTTTGTGCTGTGATCTGTCC	1.6
RNaP_FIP	ATAGCCCTCCTAGGCTCCTTTTTCCCTCTATCTGCAACTTG	1.6
N2_B3	ACTTGATCTTTGAAATTTGGATCT	0.2
N2_F3	ACCAGGAACTAATCAGACAAG	0.2
N2_LB	CTTCGGGAACGTGGTTGACC	0.4
N2_LF	GGGGCAAATTGTGCAATTTG	0.4
N2_BIP	CGCATTGGCATGGAAGTCACAATTTGATGGCACCTGTGTA	1.6
N2_FIP	CCGAAGAACGCTGAAGCGGAACTGATTACAAACATTGGCC	1.6
E1_B3	TCAGATTTTTAACACGAGAGT	0.2
E1_F3	TGAGTACGAACTTATGTACTCAT	0.2
E1_LB	CGCTTCGATTGTGTGCGT	0.4
E1_LF	CGCTATTAACTATTAACG	0.4
E1_BIP	TGCTAGTTACACTAGCCATCCTTAGGTTTTACAAGACTCACG	1.6
E1_FIP	ACCACGAAAGCAAAAAGAAAGTTCGTTTCGGAAGAGACAG	1.6
ACTB_B3	AGCCTGGATAGCAACGTACA	0.2
ACTB _F3	AGTACCCCATCGAGCACG	0.2
ACTB _LB	GAGAAGATGACCCAGATCATGT	0.4
ACTB _LF	GTGGTGCCAGATTTTCTCCA	0.4
ACTB _BIP	AGCCACACGCAGCTCATTGTATCACCAACTGGGACGACA	1.6
ACTB _FIP	CTGAACCCCAAGGCCAACCGGCTGGGGTGTTGAAGGTC	1.6

Table S4. In silico analysis of mutation frequencies within primers and probes.

Primer	Mutation (frequency)* SARS Cov-2 sequences collected between 21.3. - 20.4.2021 (n=53251)	Mutation (frequency)* SARS Cov-2 sequences collected between 7.9.-7.10.2021 (n=47807)
N2_3B	G29300C (0.16%), C29311T(0.1%)	C29330T (0.29%), C29347A (0.12%)
N2_3F	C29149T (0.12%)	T29164A (0.13%), C29160G (0.13%)
N2_LB	C29253T (0.15%), G29254 (0.13%)	C29274A (2.64%), T29283C(0.48%), A29275C(0.41%), C29271T(0.3%), A29282(0.22%)
N2_LF	<0.1%	<0.1%
N2_BIP	C29247T (0.11%), C29272T (0.56%), C29284 (0.29%)	T29252C(0.25%), A29292C(0.16%), G29300T(0.14%)
N2_FIP	C29171T (0.19%), C29218T (0.11%)	C29189T (0.35%), A29188G (0.29%), T29193G (0.15%)
E1_3B	<0.1%	T26442G(0.47%), G26450C(0.14%)
E1_3F	<0.1%	<0.1%
E1_LB	<0.1%	<0.1%
E1_LF	<0.1%	<0.1%
E1_BIP	<0.1%	<0.1%
E1_FIP	C26256T (0.26%), C26270T (0.2%), C26313T (0.11%), T26307A (0.11%)	A26271C (0.18%)
nSCoV_N1_F	A28295G (0.28%), C28291T (0.1%)	C28311G (19.99%), A28304T(0.34%), G28314C(0.13%)
nSCoV_N1_R	G28357T (0.11%)	G28362A (0.2%)
nSCoV_N1_P	C28310T (0.51%), C28313T (0.37%), C28312T (0.2%), C28311T (0.18%), G28321T (0.16%)	A28343T (0.17%)
nSCoV_N2_F	C29171T (0.19%), G29179T (0.14%)	C29200A (0.37%), C29189T(0.35%), A29188G(0.19%), T29193G(0.15%)
nSCoV_N2_R	C29218T (0.11%)	G29239A(0.19%), C29245T(0.14%), T29244G(0.12%)
nSCoV_N2_p	C29200T (0.28%)	T29225G (0.2%)
nSCoV_S_F	C21727T (0.38%), A21717G (0.29%)	T21737- (0.34%), T21733C(0.32%), C21736T(0.14%), T21738G(0.11%)
nSCoV_S_R	A21801C (1.21%), A21817G (0.25%)	T21818G (0.37%), G21809T(0.3%)
RdRP_SARSr-F	G15439A (0.23%), G15451A (0.12%)	C15455T(99.84%)
RdRP_SARSr-R	<0.1%	<0.1%
RdRP_SARSr-P2	<0.1%	<0.1%
RdRP_SARSr-P1	<0.1%	<0.1%
E_Sarbeco_F	C26270T (0.19%)	<0.1%
E_Sarbeco_R	<0.1%	<0.1%
E_Sarbeco_P1	<0.1%	<0.1%

*only shown mutations with frequencies ≥0.1%

Numbers in red indicate significant increase in mutation frequency.

Table S5. Performance of the SARS-CoV-2 1–step RT-LAMP and RT-qPCR for different saliva specimens.

**RT-LAMP**	**Saliva / NPS***	
N2 (LAMP-2/20)	Sensitivity	Specificity
Rayon#	3/30 (10 %)	51/55 (93 %)
Nylon#	52/141 (37 %)	342/355 (96 %)
Passive drool#	65/104 (63%)	57/58 (98%)
**RT-qPCR method**	**Saliva / NPS***	
N1 (S-2/20)	Sensitivity	Specificity
Rayon#	14/30 (47 %)	53/55 (96 %)
Nylon#	123/142 (87 %)	353/358 (99 %)
Passive drool#	99/104 (95%)	86/86 (100%)

All saliva specimens were positive for human RNaP RNA
* paired NPS and saliva specimens.

Table S6. Implementation of the SARS-CoV-2 1-step RT-LAMP test for passive drool saliva.

**RT-LAMP**	**Passive drool compared to NPS***		
	N2	E1	N2 or E1
(LAMP-2/20)#	65/104 (63%)	-	-
Cq<20	17/23 (74%)	-	
Cq 20-25	28/41 (68%)	-	
Cq 25-30	10/21 (48%)	-	
Cq>30	10/19 (53%)	-	
(LAMP-5/15)&	76/103 (74%)	72/103 (70%)	85/103 (83%)
Cq<20	20/23 (87%)	19/23 (83%)	
Cq 20-25	30/40 (75%)	27/40 (68%)	
Cq 25-30	15/21 /71%)	13/21 (62%)	
Cq>30	11/19 (58%)	13/19 (68%)	

#,& all saliva specimens were positive for human RNaP RNA or 18S rRNA.
* paired NPS and saliva specimens.

Table S7. Performance of the SARS-CoV-2 1-step RT-qPCR test for passive drool saliva.

**RT-qPCR**	**Passive drool compared to NPS***			
	**Diagnostic sensitivity**			**Specificity**
	N1	N2	N1 or N2	
Singleplex				
(S-2/20)#	99/104 (95%)	-	-	86/86 (100%)
Cq<20	23/23 (100%)	-	-	
Cq 20-25	39/41 (95%)	-	-	
Cq 25-30	20/21 (95%)	-	-	
Cq>30	17/19 (89%)	-	-	
Multiplex				
(M-5/20)#	95/101 (94%)	92/101 (91%)	96/101 (95%)	not determine
(M-2.5/10)#	97/101 (96%)	94/101 (93%)	99/101 (98%)	not determine
Singleplex				
(S-5/15)&	89/102 (88%)	91/102 (89%)	98/102 (96%)	80/81 (99%)
Cq<20	21/23 (91%)	22/23 (96%)		
Cq 20-25	37/40 (93%)	36/40 (90%)		
Cq 25-30	18/20 (90%)	19/20 (95%)		
Cq>30	15/19 (79%)	14/19 (74%)		

#,& all saliva specimens were positive for human RNaP RNA or 18S RNA.
* paired NPS and saliva specimens.

Table S8. SARS-CoV-2 mass testing of healthy individuals and screening of COVID-19 patients.

	**RT-LAMP**	**RT-qPCR***	**Combined RT-LAMP or RT-qPCR**
Healthy individuals	769	9	769
Positive	33/35 (94%)	9/9 (100%)	33/35 (94%)
Negative	720/734 (98%)	-	720/734 (98%)
COVID-19 patients	79	44	79
Positive	40/78 (51%)	26/43 (60%)	52/78 (67%)
Negative	1	1	1 (100%)

*Saliva-based one-step RT-qPCR was used to confirm some RT-LAMP results.
